# End-Tagging of Ultra-Short Antimicrobial Peptides by W/F Stretches to Facilitate Bacterial Killing

**DOI:** 10.1371/journal.pone.0005285

**Published:** 2009-04-17

**Authors:** Mukesh Pasupuleti, Artur Schmidtchen, Anna Chalupka, Lovisa Ringstad, Martin Malmsten

**Affiliations:** 1 Section of Dermatology and Venereology, Department of Clinical Sciences, Lund University, Lund, Sweden; 2 Department of Pharmacy, Uppsala University, Uppsala, Sweden; Cinvestav, Mexico

## Abstract

**Background:**

Due to increasing resistance development among bacteria, antimicrobial peptides (AMPs), are receiving increased attention. Ideally, AMP should display high bactericidal potency, but low toxicity against (human) eukaryotic cells. Additionally, short and proteolytically stable AMPs are desired to maximize bioavailability and therapeutic versatility.

**Methodology and Principal Findings:**

A facile approach is demonstrated for reaching high potency of ultra-short antimicrobal peptides through end-tagging with W and F stretches. Focusing on a peptide derived from kininogen, KNKGKKNGKH (KNK10) and truncations thereof, end-tagging resulted in enhanced bactericidal effect against Gram-negative *Escherichia coli* and Gram-positive *Staphylococcus aureus*. Through end-tagging, potency and salt resistance could be maintained down to 4–7 amino acids in the hydrophilic template peptide. Although tagging resulted in increased eukaryotic cell permeabilization at low ionic strength, the latter was insignificant at physiological ionic strength and in the presence of serum. Quantitatively, the most potent peptides investigated displayed bactericidal effects comparable to, or in excess of, that of the benchmark antimicrobial peptide LL-37. The higher bactericidal potency of the tagged peptides correlated to a higher degree of binding to bacteria, and resulting bacterial wall rupture. Analogously, tagging enhanced peptide-induced rupture of liposomes, particularly anionic ones. Additionally, end-tagging facilitated binding to bacterial lipopolysaccharide, both effects probably contributing to the selectivity displayed by these peptides between bacteria and eukaryotic cells. Importantly, W-tagging resulted in peptides with maintained stability against proteolytic degradation by human leukocyte elastase, as well as staphylococcal aureolysin and V8 proteinase. The biological relevance of these findings was demonstrated *ex vivo* for pig skin infected by *S. aureus* and *E. coli*.

**Conclusions/Significance:**

End-tagging by hydrophobic amino acid stretches may be employed to enhance bactericidal potency also of ultra-short AMPs at maintained limited toxicity. The approach is of general applicability, and facilitates straightforward synthesis of hydrophobically modified AMPs without the need for post-peptide synthesis modifications.

## Introduction

Due to increasing resistance development among bacteria, antimicrobial peptides (AMPs), are currently receiving increased attention [1]–[7]. Ideally, AMP should display high bactericidal potency, but low toxicity against (human) eukaryotic cells. Approaches to reach such selectivity include QSAR in combination with directed amino acid modifications, as well as identification of AMPs of endogenous origin [8]–[19]. Since bacterial membrane rupture is a key mechanism of action of these peptides, AMPs should bind to, and rupture, bacterial membranes but not eukaryotic membranes. Due to bacterial membranes being cholesterol-void and dominated by anionic phospholipids, whereas (human) eukaryotic membranes contain cholesterol and are dominated by zwitterionic ones, some AMP binding selectivity can be obtained for positively charged and hydrophilic AMPs [20]. However, the electrostatic surface potential of *S. aureus* and some other common pathogens is frequently limited, and may be reduced or even reversed, e.g., by L-lysine modification of phosphatidylglycerol, D-alanine modification of cell wall teichoic acid, and aminoarabinose modifications in LPS, all precluding AMP binding [4]. Additionally, electrostatically driven peptide binding is salt sensitive, and bactericidal potency of such peptides at physiological ionic strength limited. This situation can be remedied by increasing the hydrophobicity of AMPs, although AMPs of higher hydrophobicity have been found to be less selective in their action, and to display increased toxicity [21]. Given the above, and inspired by lipopeptides [22]–[27], we previously identified end-tagging of AMPs with hydrophobic amino acid stretches as a facile and readily tunable approach to achieve high adsorption of partially submerged, highly charged AMPs [28]. Such end-tagged peptides were found to display limited toxicity combined with high microbicidal potency of broad spectrum, also at physiological ionic strength and in the presence of serum, as well as *ex vivo* and *in vivo*. From detailed physico-chemical investigations, involving studies on peptide adsorption at supported lipid bilayers, peptide-induced liposome rupture, both as a function of peptide sequence, electrolyte concentration, and lipid membrane composition, as well as LPS-binding experiments, circular dichroism experiments on peptide conformation, and studies on bacterial wall rupture, it was concluded that the end-tagged peptides reach their potency and salt resistance through the hydrophobic end-tags promoting peptide adsorption at phospholipid membranes. The selectivity between bacteria and eukaryotic cells could also be explained on a mechanistic level, and due to the lower charge density of eukaryotic cell membrane, combined with the presence of cholesterol in the latter. Through the membrane-condensing effect of cholesterol, incorporation of bulky end-tags (shown to take place in the polar headgroup region of the phospholipid membrane) is precluded, resulting in lower membrane incorporation and rupture, and contributing to the selectivity observed between bacteria and eukaryotic cells.

In the present study, we bring this work further by investigating whether tagging by hydrophobic amino acid stretches may be employed to enhance bactericidal potency also of ultra-short AMPs at maintained limited toxicity. This is a key aspect for the wider therapeutic use of AMPs, since the macromolecular nature of AMPs, typically containing 20–40 amino acids, limits their use through administration routes other than the topical and parenteral ones, and precludes their biological uptake, e.g., by the gastrointestinal, nasal, transdermal, and pulmonary routes. This precludes the wider pharmaceutical applicability of longer AMPs. If AMP potency and selectivity can be retained for shorter peptides, on the other hand, a range of potential new indications for AMP therapies opens up related to their higher and wider bioavailability [29].

## Materials and Methods

### Peptides

The high quality peptides used in this work were synthesized by Biopeptide Co., San Diego, USA, with the exception of LL-37 (LLGDFFRKSKEKIGKEFKRIVQRIKDFLRNLVPRTES), which was obtained from Innovagen AB, Lund, Sweden. The purity (>95%) of these peptides was confirmed by mass spectral analysis (MALDI-ToF Voyager), provided by the suppliers. Peptides for the initial screening were from Sigma-Genosys (Sigma-Aldrich, St. Louis, USA), generated by a peptide synthesis platform (PEPscreen®, Custom Peptide Libraries, Sigma Genosys) with a yield of ∼1–6 mg. MALDI-ToF Mass Spectrometry was performed on these peptides, and average crude purity found to be 60–70%. Prior to biological testing the PEPscreen peptides were diluted in H_2_0 (5 mM stock), and stored at −20 C. This stock solution was used for the subsequent experiments.

### Microorganisms

*Escherichia coli* ATCC 25922 and *Staphylococcus aureus* ATCC 29213 were obtained from the Department of Clinical Bacteriology at Lund University Hospital.

### Radial diffusion assay (RDA)

Essentially as described earlier [30], [31] bacteria were grown to mid-logarithmic phase in 10 ml of full-strength (3% w/v) trypticase soy broth (TSB) (Becton-Dickinson, Cockeysville, USA). The microorganisms were then washed once with 10 mM Tris, pH 7.4. Subsequently, 4×10^6^ bacterial colony forming units were added to 15 ml of the underlay agarose gel, consisting of 0.03% (w/v) TSB, 1% (w/v) low electroendosmosis type (EEO) agarose (Sigma-Aldrich, St. Louis, USA) and 0.02% (v/v) Tween 20 (Sigma-Aldrich, St. Louis, USA). The underlay was poured into a Ø 144 mm petri dish. After agarose solidification, 4 mm-diameter wells were punched and 6 µl of peptide with required concentration added to each well. Plates were incubated at 37°C for 3 hours to allow diffusion of the peptides. The underlay gel was then covered with 15 ml of molten overlay (6% TSB and 1% Low-EEO agarose in distilled H_2_O). Antimicrobial activity of a peptide is visualized as a zone of clearing around each well after 18–24 hours of incubation at 37°C. Results given represent mean values from triplicate measurements.

### Protease sensitivity assay

Peptides (1 µg) were incubated at 37°C with *S. aureus* aureolysin (0.1 µg, 25000 units/mg), *S. aureus* V8 proteinase (0.1 µg, 2000 mU), both from BioCol GmbH (Potsdam, Germany), or neutrophil elastase (0.4 µg, 29 units/mg; Calbiochem (La Jolla, USA)) in a total volume of 30 µl for 3 hours. The materials were analyzed on 16.5% precast sodium dodecyl sulfate polyacrylamide (SDS-PAGE) Tris-Tricine gels (BioRad, Hercules, USA) and analyzed after staining with Coomassie Blue R-250 (Merck, Darmstadt, Germany).

### MTT assay

Sterile filtered MTT (3-(4,5-dimethylthiazolyl)-2,5-diphenyl-tetrazolium bromide; Sigma-Aldrich, St. Louis, USA) solution (5 mg/ml in PBS) was stored protected from light at −20°C until usage. HaCaT keratinocytes, 3000 cells/well, were seeded in 96 well plates and grown in keratinocyte-SFM/BPE-rEGF medium to confluence. Keratinocyte-SFM/BPE-rEGF medium alone, or keratinocyte-SFM supplemented with 20% serum, was added, followed by peptide addition to 60 µM. After incubation over night, 20 µl of the MTT solution was added to each well and the plates incubated for 1 h in CO_2_ at 37°C. The MTT- containing medium was then removed by aspiration. The blue formazan product generated was dissolved by the addition of 100 µl of 100% DMSO per well. The plates were then gently swirled for 10 min at room temperature to dissolve the precipitate. The absorbance was monitored at 550 nm, and results given represent mean values from triplicate measurements.

### Lactate dehydrogenase (LDH) assay

HaCaT keratinocytes were grown in 96 well plates (3000 cells/well) in serum-free keratinocyte medium (SFM) supplemented with bovine pituitary extract and recombinant EGF (BPE-rEGF) (Invitrogen, Eugene, USA) to confluency. The medium was then removed, and 100 µl of the peptides investigated (at 60 µM, diluted in SFM/BPE-rEGF or in keratinocyte-SFM supplemented with 20% human serum) added in triplicates to different wells of the plate. The LDH-based TOX-7 kit (Sigma-Aldrich, St. Louis, USA) was used for quantification of LDH release from the cells. Results given represent mean values from triplicate measurements, and are given as fractional LDH release compared to the positive control consisting of 1% Triton X-100 (yielding 100% LDH release).

### Hemolysis assay

EDTA-blood was centrifuged at 800 g for 10 min, whereafter plasma and buffy coat were removed. The erythrocytes were washed three times and resuspended to 5% in PBS, pH 7.4. For experiments in 50% blood, EDTA-blood was diluted (1∶1) with PBS. The cells were then incubated with end-over-end rotation for 1 h at 37°C in the presence of peptides (60 µM). 2% Triton X-100 (Sigma-Aldrich, St. Louis, USA) served as positive control. The samples were then centrifuged at 800 g for 10 min. The absorbance of hemoglobin release was measured at 540 nm and is expressed as % of TritonX-100 induced hemolysis. Results given represent mean values from triplicate measurements.

### Slot-blot assay

LPS and heparin binding ability of the peptides were examined by slot-blot assay. Peptides (1, 2 and 5 µg) were bound to nitrocellulose membrane (Hybond-C, GE Healthcare BioSciences, Buckinghamshire, UK), pre-soaked in PBS, by vacuum. For LPS binding, membranes were then blocked by 2 wt% BSA in PBS, pH 7.4, for 1 h at RT and subsequently incubated with radiolabelled LPS (40 µg/mL; 0.13×10^6^ cpm/µg) for 1 h at RT in PBS [32]. For heparin binding, the peptide-loaded membrane was incubated with radiolabelled heparin (10 µg/mL; 0.2×10^6^ cpm/µg) for 1 h in PBS [33], without prior BSA blocking. The radioiodination (^125^I) of heparin and LPS was performed as described earlier [32]. After LPS and heparin binding, the membranes were washed 3 times, 10 min each time, in PBS and visualized for radioactivity on Bas 2000 radioimaging system (Fuji, Tokyo, Japan).

### Antibacterial effects *ex vivo*

For evaluating AMPs *ex vivo*, a pig skin model was used as previously described [34] but with modifications. Defatted pig hides were first washed with water and then with 70% ethanol. They were then destubbled with disposable razors and 8×8 cm pieces were cut, sealed in plastic wrap, and frozen at −20°C. Before use, skin samples were thawed, washed with ethanol (70%) and water. In order to separate the inoculation areas, sterilized tubings (polyethylene, 9.6 m, Nalgene® VWR 228-0170) were cut into ∼10 mm lengths, and glued onto the skin samples (cyanoacrylate glue, Henkel, Düsseldorf, Germany). The skin was infected by adding 1×10^6^ CFU of an overnight culture of *S. aureus* ATCC 29213 and *E. coli* ATCC 25922 in a total volume of 10 µl. After an incubation time of 4 hours at 37°C, peptide-containing solutions (1 mM) were applied. Bacterial sampling was performed after an incubation time of 2 hours by washing the reaction chambers twice with 250 µl of 10 mM phosphate buffer, pH 7.4, 0.05wt% Triton X-100, supplemented with 0.1% dextran sulfate, added to block peptide activity during sampling (average molecular weight 500 kDa, Sigma-Aldrich, St. Louis, USA). Results given represent mean values (n = 6).

### Liposome preparation and leakage assay

The liposomes investigated were either zwitterionic (DOPC) or anionic (either DOPE/DOPG 75/25 mol/mol or *E. coli* lipid extract containing 57.5% phosphatidylethanolamine, 15.1% phosphatidylglycerol, 9.8% cardiolipin, and 17.6% other lipids). DOPG (1,2-Dioleoyl-sn-Glycero-3-Phosphoglycerol, monosodium salt), DOPC (1,2-dioleoyl-sn-Glycero-3-phoshocholine), and DOPE (1,2-dioleoyl-sn-Glycero-3-phoshoetanolamine) were all from Avanti Polar Lipids (Alabaster, USA) and of >99% purity. Due to the long, symmetric and unsaturated acyl chains of the pure phospholipids, several methodological advantages are reached. In particular, membrane cohesion is good, facilitating very stable and unilamellar liposomes (observed from cryo-TEM), and allowing precise values on leakage to be obtained. The lipid mixtures were dissolved in chloroform, whereafter the solvent was removed by evaporation under vacuum overnight. Subsequently, 10 mM Tris buffer, pH 7.4, was added together with 0.1 M carboxyfluorescein (CF) (Sigma, St. Louis, USA). After hydration, the lipid mixture was subjected to eight freeze-thaw cycles (not the *E. coli* lipid mixture) consisting of freezing in liquid nitrogen and heating to 60°C. In all cases, unilamellar liposomes of about Ø140 nm were generated by multiple extrusions through polycarbonate filters (pore size 100 nm) mounted in a LipoFast miniextruder (Avestin, Ottawa, Canada) at 22°C. Untrapped CF was then removed by two subsequent gel filtrations (Sephadex G-50, GE Healthcare, Uppsala, Sweden) at 22°C, with Tris buffer as eluent. CF release from the liposomes was determined by monitoring the emitted fluorescence at 520 nm from a liposome dispersion (10 mM lipid in 10 mM Tris, pH 7.4). An absolute leakage scale was obtained by disrupting the liposomes at the end of each experiment through addition of 0.8 mM Triton X-100 (Sigma-Aldrich, St. Louis, USA). A SPEX-fluorolog 1650 0.22-m double spectrometer (SPEX Industries, Edison, USA) was used for the liposome leakage assay. Measurements were performed in triplicate at 37°C.

### Statistics

Values are reported as means±standard deviation of the means. To determine significance, analysis of variance with ANOVA (SigmaStat, SPSS Inc., Chicago, USA), followed by *post hoc* testing using the Holm-Sidak method, was used as indicated in the figure legends, where “n” denotes number of independent experiments. Significance was accepted at p<0.05.

## Results

**Figure 1:**
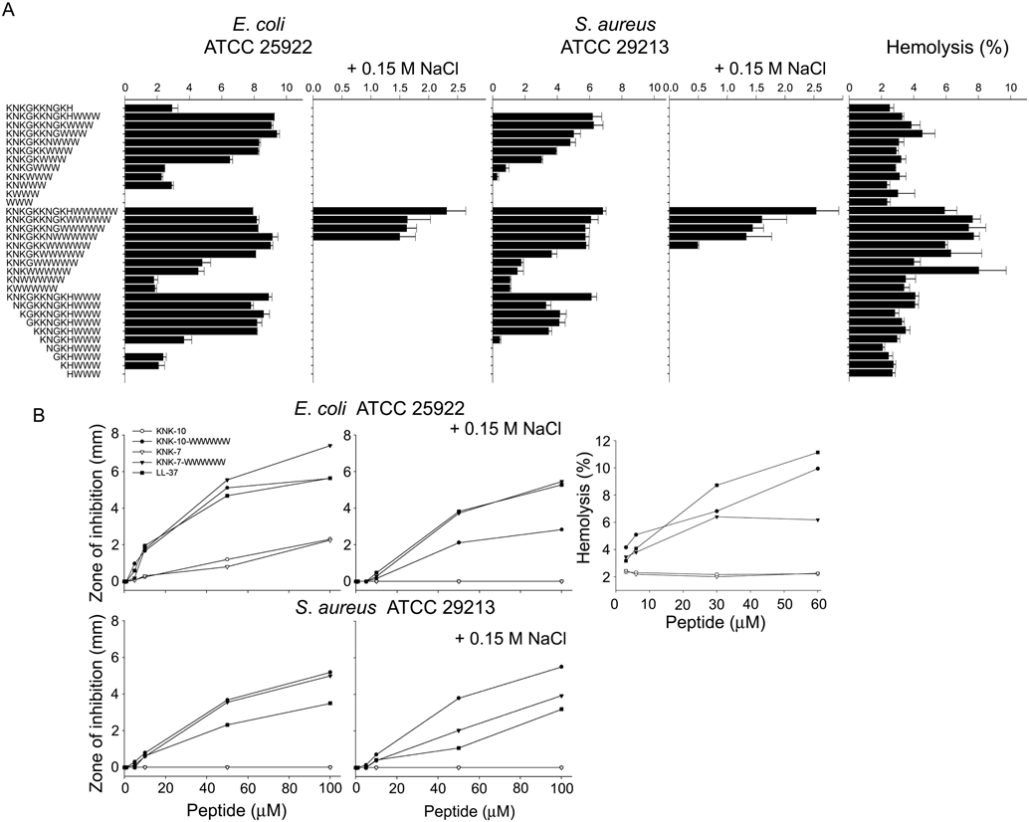
Antibacterial and hemolytic activity of peptides. (A) Antibacterial activity of PEPscreen peptides as assessed by radial diffusion assay (RDA) in the presence and absence of 0.15 M NaCl against *E. coli* ATCC 25922 and *S. aureus* ATCC 29213, as well as hemolysis. For determination of antibacterial activity, bacteria (4×10^6^ cfu/ml) were inoculated in 0.1% TSB agarose gel and 4 mm wells punched. In each well 6 µl of peptide (at 100 µM) was loaded. The zones of clearance correspond to the inhibitory effect of each peptide after incubation at 37°C for 18–24 h (mean values are presented, n = 3). For hemolysis, the cells were incubated with the peptides at 60 µM, while 2% Triton X-100 served as positive control. The absorbance of hemoglobin release was measured at 540 nm and is expressed as % of Triton X-100 induced hemolysis. (B) Dose dependent activity of selected high purity peptides KNK10, KNK7, and W-modified variants of these against E. *coli* ATCC 25922 and *S. aureus* ATCC 29213 in RDA. Shown also are effects of the peptides on human erythrocytes in the hemolysis assay. Throughout, mean values are presented, n = 3. (In Figure 1B, the difference between tagged and non-tagged peptides is statistically significant in all cases (P<0.001, two way ANOVA).) WWW tripeptide alone yielded non-measurable bactericidal effects (0 mm clearance zone in RDA) under the conditions used in Figure 1A, as well as hemolysis (2.3±0.2%) comparable to that in the negative control (2.5±0.05%). Due to poor aqueous solubility, longer oligotryptophan peptides could not be investigated.

As an initial step, effects of W-tag length, as well as hydrophilic peptide length and composition, on the bactericidal potency were investigated by RDA for Gram-negative *E. coli* and Gram-positive *S. aureus*. For both bacteria, tagging of KNK10 by either WWW or WWWWW significantly increases bactericidal potency, also at high ionic strength (Figure 1A). Truncating KNK10 from either C- or N-terminus results in a decrease in both bactericidal potency and salt resistance, although very short peptides are reached in both series (KNK5-WWW, KNK4-WWWWW, and KNG5-WWW, respectively) before significant reduction in bactericidal potency is observed at low ionic strength. At high ionic strength, substantial bactericidal potency was observed for the longer WWWWW tag down to KNK7-WWWWW. The WWW tag, on the other hand, is too short to provide bactericidal potency at high ionic strength when combined with the short hydrophilic peptide stretches.

Bactericidal potency was probed also for selected peptides of high purity: KNK7, KNK10, KNK7-WWWWW, and KNK10-WWWWW (Figure 1B). Neither non-tagged KNK7 nor KNK10 displayed substantial bactericidal activity against *E. coli* and *S. aureus.* End-tagging either of these peptides with WWWWW, on the other hand, resulted in strongly bactericidal peptides against both *E. coli* and *S. aureus*, also at high ionic strength. Quantitatively, the bacterial killing is more potent for KNK10-WWWWW than for KNK7-WWW, with bactericidal potency comparable to that of the benchmark peptide LL-37. Similar effects were found also for F-tagged peptides (Figure S1A).

**Figure 2:**
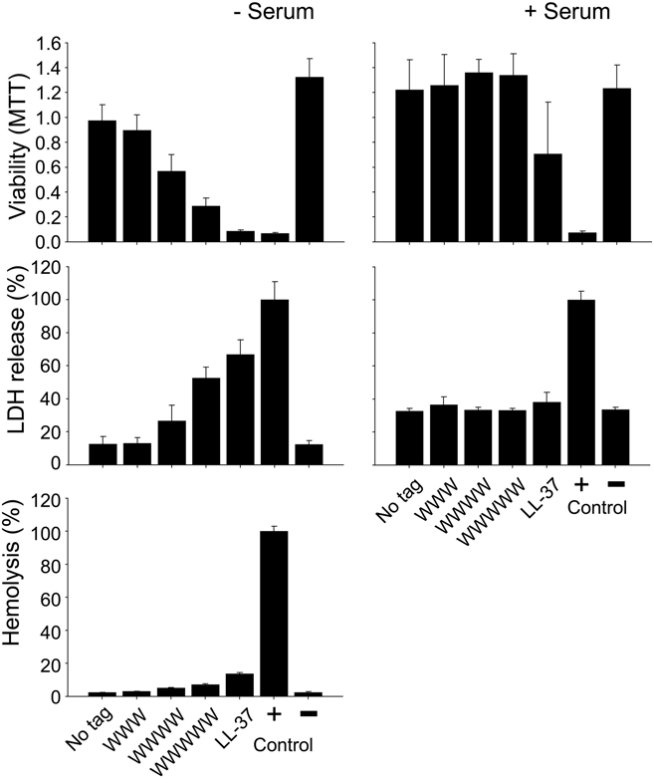
Effect of peptides on eukaryotic cells. Effects of peptides on HaCaT cells and erythrocytes in absence and presence of 20% human serum. The MTT-assay (upper panel) was used to measure viability of HaCaT keratinocytes in the presence of KNK10 peptides with variable W tagging. In the assay, MTT is modified into a dye, blue formazan, by enzymes associated to metabolic activity. The absorbance of the dye was measured at 550 nm. Cell permeabilizing effects of the indicated peptides (middle panel) were measured by the LDH-based TOX-7 kit. Hemolytic effects of the indicated peptides are also shown (lower panel). Cells were incubated with peptides at 60 µM, while 2% Triton X-100 served as positive control. The absorbance of hemoglobin release was measured at 540 nm and is expressed as % of Triton X-100 induced hemolysis (mean values are presented, n = 3). (For MTT and LDH, the difference between tagged and non-tagged peptides is statistically significant in the absence of serum (P<0.001, one way ANOVA), whereas the difference in the presence of serum is not statistically significant. For hemolysis, the difference between tagged and non-tagged peptides is not statistically significant.)

The non-tagged peptides show no hemolysis above that of the negative control (Figure 1 and Figure 2. Tagging with WWW results in little, if any, increase in hemolysis, whereas that of the longer WWWWW results in a slightly increased hemolysis. This effect is concentration dependent, with hemolysis for KNK10-WWWWW being comparable to, or lower than, that of LL-37 (Figure 1B). Similarly, tagging with WWWWW, but not WWW, results in an increased cell permeabilization monitored by MTT and LDH assays. In the presence of serum, on the other hand, also the WWWWW-tagged peptide displays no detectable permeabilization with either LDH release or MTT assay (Figure 2). Again, similar results were obtained for the F-tagged peptides (Figure S1B).

**Figure 3:**
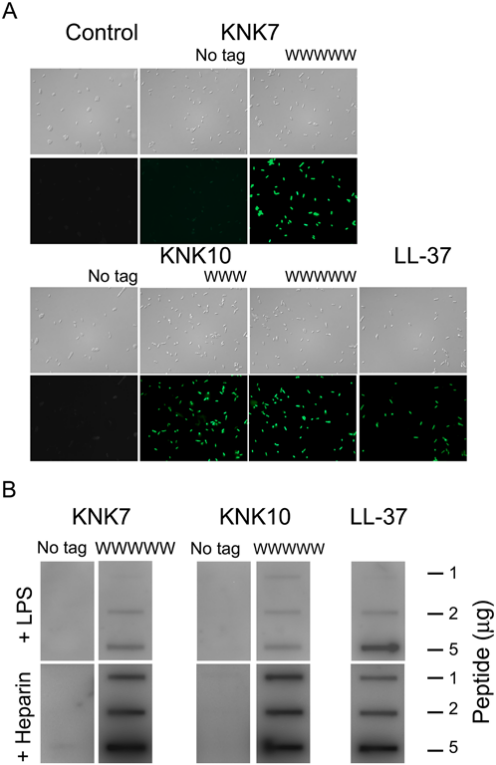
Peptide interaction with bacteria and LPS. (A) Permeabilizing effects of peptides on bacteria. *E. coli* was incubated with KNK7, KNK10, and the indicated W-modified variants (all at 30 µM) in buffer at physiological salt (0.15 M NaCl) for 2 h at 37°C, after which permeabilization was assessed using the impermeant probe FITC. The upper images in each row are Nomarski Differential Interference Contrast images, while the lower show FITC fluorescence of bacteria. (B) LPS and heparin binding abilities of the KNK7, KNK10 and the indicated W-modified variants peptides.

**Figure 4:**
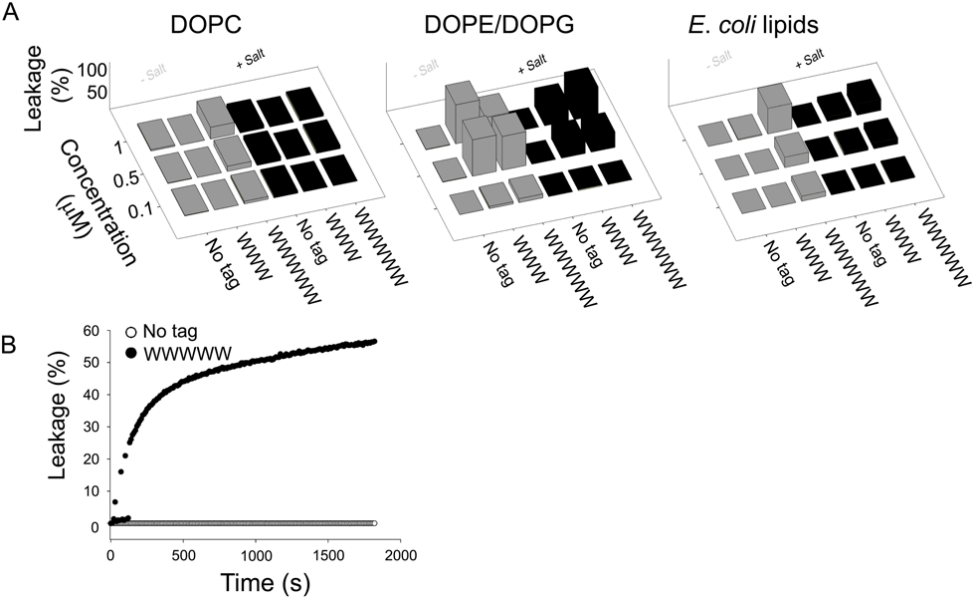
Peptide-mediated permeabilization of liposomes. (A) Effects of KNK10 and indicated W-modified peptides on liposomes in the presence and absence of 0.15 M NaCl. The membrane permeabilizing effect, and resulting release of carboxyfluorescein from liposomes, was recorded by fluorescence spectroscopy. Left panel shows DOPC (zwitterionic) liposomes, the center panel DOPE/ DOPG (75/25 mol/mol; anionic) liposomes, and the right panel (anionic) liposomes formed by *E. coli* lipids (mean values are presented, n = 3). (B) Leakage kinetics on addition of 1 µM KNK10 and KNK10-WWWWW to DOPE/DOPG (75/25 mol/mol; anionic) liposomes in 10 mM Tris buffer, pH 7.4, with 150 mM NaCl.

As can be seen in Figure 3A, the increased bactericidal potency of the W-tagged peptides correlates to a higher permeabilization of bacteria. In analogy, results from anionic liposomes composed of either a bacteria-mimicking lipid mixture (DOPE/DOPG) or lipid extract from *E. coli*, showed tagged peptides to be much more potent in causing membrane rupture and liposome leakage that the corresponding non-tagged ones (Figure 4A). For both these lipid mixtures, rapid and extensive leakage induction was observed with the tagged peptides (Figure 4B). As with bacterial killing, peptide-induced liposome leakage increased with increasing peptide concentration and hydrophobic tag length, and was partially reduced at high ionic strength, the salt inactivation decreasing with increasing hydrophobic tag length. In analogy to the bactericidal and cytotoxicity results, liposome leakage induction by the tagged peptides is substantial for both negatively charged (“bacterial”) lipid mixtures investigated, but substantially lower for zwitterionic (“eukaryotic”) DOPC liposomes, particularly at high ionic strength. Additionally, W-tagging was found to facilitate binding of both KNK10 and KNK7 to LPS (and heparin) also at high ionic strength (Figure 3B), an effect which can be completely reversed through addition of heparin, acting as an anionic competitor to LPS for the tagged peptides (results not shown).

**Figure 5:**
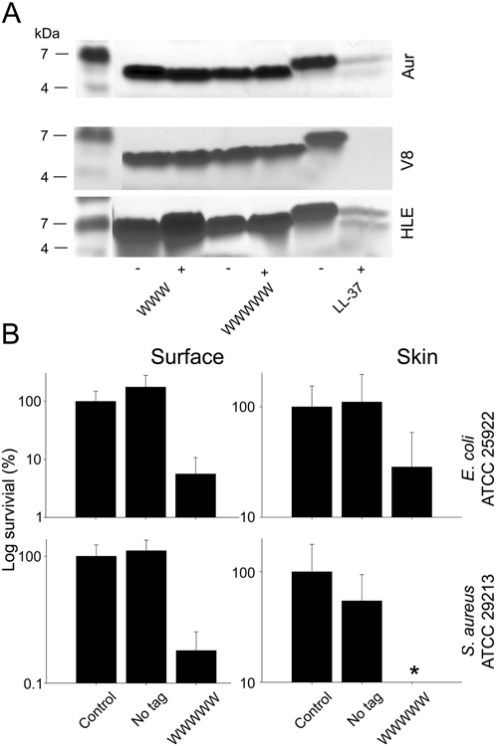
(A) Protease sensitivity of peptides. KNK10 and KNK10-WWWWW were incubated with (+) or without (−) the *S. aureus* enzymes aureolysin (Aur), V8 proteinase (V8), or human leukocyte elastase (HLE), and analyzed by SDS-PAGE (16.5% Tris-Tricine gels). (B) *Activities of peptides in an ex vivo skin infection model.* Pig skin was inoculated with *E. coli* ATCC 25922 (upper panel) or *S. aureus* ATCC 29213 (lower panel). Peptides at 1 mM were added after an incubation time of 4 h. Bacteria were collected after 2 h and cfu determined (mean values are presented, n = 6). Note the logarithmic scale on the y-axis. (In Figure 5B, the difference between tagged and non-tagged peptide is statistically significant in all cases (P<0.002, one way ANOVA).)

Since one of the attractive features of KNK10 is its relative stability against proteolytic degradation, we also investigated if hydrophobic tagging effected the peptide proteolytic stability. Similar to KNK10, KNK10-WWWWW displayed good stability against proteolytic degradation by human leukocyte elastase, as well as staphylococcal aureolysin and V8 proteinase (Figure 5A). (Similar results were obtained also with KNK10-FFFFF and KNK7-WWWWW (results not shown)). In contrast, but in agreement with previous findings [28], [35], LL-37 undergoes substantial degradation by all these enzymes. From this it is clear that the tagging can be achieved at maintained proteolytic stability of the peptide.

*S. aereus* is a notorious pathogen in relation to a number of skin infections, including atopic dermatitis, impetigo, and wound infections [36]. Frequently, the spread of the infection is mediated by bacterial proteases, which degrade both collagen and non-collagen host proteins, thus destroying host physical carriers. Hence, the stability of the end-tagged peptides, notably KNK10-WWWWW, against a range of proteases, combined with potent bactericidal effects, could make this peptide a potential candidate for skin infection therapies. In order to demonstrate this, the effect of KNK10-WWWWW was investigated in a skin wound model. For both *E. coli*, which sometimes contaminates wounds and causes surgical site infections [37], and *S. aureus*, KNK10-WWWWW, but not the non-tagged KNK10, drastically reduced bacteria viability at the skin surface (Figure 5B, left panel). Although quantitatively smaller effects were observed deeper down in the skin, KNK10-WWWWW nevertheless caused significant reduction of both bacteria investigated, while the non-tagged KNK10 was much less potent (Figure 5B, right panel).

## Discussion

Although AMPs influence bacteria in a multitude of ways, e.g., DNA binding, enzyme activities, and cell wall synthesis [5], their main mode of action is defect formation in bacterial walls, notably its lipid membrane(s) [1]–[7]. For some peptides, e.g., melittin, alamethicin, gramicidin A, and magainin 2, this is achieved through the formation of transmembrane structures, sometimes associated with induction of an ordered secondary structure, notably α-helix structures [5], [6], [38]. For disordered peptides with a high net charge, membrane distruption is reached by other mechanisms, e.g., induction of a negative curvature strain, membrane thinning, or local packing defect formation associated with peptide localization primarily in the polar headgroup region of the phospholipids membrane [5], [20], [39]–[42]. In the latter case, defect formation increases with the amount of peptide adsorbed at the lipid membrane and with the peptide charge [20], [39], [40]. Although a high positive peptide net charge may result in high adsorption of AMPs to highly negatively charged bacterial membranes, electrostatic interactions are screened at high ionic strength, resulting in reduced driving force for AMP adsorption, and in partial or complete loss in bactericidal capacity at physiological conditions. Simultaneously increasing AMP hydrophobicity may constitute a strategy for increasing salt resistanse of AMPs. Although peptides based on overall hydrophobic interactions are less selective between bacterial and eukaryotic lipid membranes, which risks resulting in an enhanced cytotoxicity of more hydrophobic AMPs [21], carefully balancing hydrophobicity and charge in a sequence-specific way may result in short, potent antimicrobial peptides displaying low mammalian cell toxicity (e.g., [43]).

Considering this, a general, facile, and tunable platform for balanced hydrophobic modifications of AMPs is desirable. Although such hydrophobic modifications can be achieved in a number of ways, end-tagging by hydrophobic amino acid stretches is one of the easier ones since it requires no post-synthesis modification, since it allows the primary AMP sequence to be retained, as well as maximized interaction between the hydrophobic tag and the phospholipid membrane. In addition, end-tagging does not detrimentally affect proteolytic stability of AMPs (Figure 5A), a factor of importance for bactericidal potency on *S. aureus*, *P. aeruginosa* and other bacteria excreting proteolytic enzymes [4].

Particularly W- and F-based ones are interesting as hydrophobic end-tags. These bulky, aromatic, and polarizable residues have an affinity to interfaces [44], [45], and are frequently located in the proximity of the polar headgroup region in phospholipids membranes [46]–[52]. Through this interaction with the phospholipids membrane, W/F residues are able to act as an anchor for the peptide, and may similarly be important for the function of other ultra-short membrane-interacting peptides, such as substance P [53]. In combination with highly charged AMP sequences, this results in an effective pinning of a large number of peptide charges in the polar headgroup region of the membrane, in turn facilitating membrane defect formation and rupture. Due to the large size of the W/F groups, combined with their surface localization, part of the selectivity between bacterial and eukaryotic cell membranes is obtained through cholesterol precluding membrane insertion of the W/F groups, and through a lower adsorption of the cationic composite peptides at zwitterionic than at anionic membranes [28]. For Gram-negative bacteria, this phospholipid membrane selectivity is accompanied by selectivity originating from LPS binding, which is significantly enhanced through the W tagging. Together, these effects probably contribute substantially to the selectivity observed with the presently investigated W/F-tagged peptides, displaying high bactericidal potency, but at the same time low toxicity.

Numerous previous studies in literature address the issue of balancing electrostatic and hydrophobic aspects for effective and selective action of various types of AMPs, including effects of hydrophobic substitutions. However, with the exception of lipopeptides, this previous work has concerned specific AMPs, for which hydrophobicity/charge variations can be said to be largely sequence-specific. The present work, as well as previous work in literature on lipopeptides, on the other hand, report on more generally applicable technology platforms of versatile use for boosting potency of AMPs. Clearly, the end-tagged peptides are somewhat related to lipopeptides, consisting of a polar (linear or cyclic, positively or negatively charged) peptide sequence with a hydrophobic moiety, e.g., a fatty acid acid, covalently attached. Lipopeptides, such as polymyxin B and colistin, are potent against particularly Gram-negative bacteria, but also display substantial toxicity [22], the latter possibly related to membrane composition-independent incorporation of acyl groups in a way comparable to that of peptides of high hydrophobicity [21]. Like antimicrobial peptides, lipopeptides affect bacteria in a multitude of ways, e.g., DNA replication, transcription, and translation, and also exert anti-endotoxic effects through LPS binding and neutralization. The latter LPS-binding effect is similar to that of the presently investigated peptides. Also in analogy to the present findings, longer acyl chains in such lipopeptides cause increased disruption of model lipid membranes, as well as activity against bacteria and fungi [22]. In contrast to lipopeptides containing long acyl chains, however, which depend on self-assembly for their bactericidal action, W/F-tags are too short, bulky, and polarizable to cause peptide self-assembly. This means that no self-assembly takes place, potentially translating to faster action since no aggregation/disintegration is needed for peptide action [28]. Of particular interest in relation to the present work is a recent investigation by Makovitzki et al., in which ultra-short peptide sequences conjugated to palmitic acid were studied [54]. In agreement with the present investigation, very short peptide sequences were found to be able to reach potency also in the biological context, although with the potency decreasing for sufficiently short hydrophilic sequences. Also in agreement with the present investigation, the antimicrobial effect of these lipopeptides was found to depend on the detailed sequence of the hydrophilic peptide stretch, and to involve membrane permeabilization and disintegration. On the other hand, the authors also observed complex and large self-assembly structures, largely driven by the long and saturated palmitoyl chains, which may affect the antimicrobial potency of these lipopeptides, particularly at temperatures below the melting temperature of palmitic acid (63–64°C) [55]. In contrast, KNK10-WWWWW and other end-tagged peptides with smaller mean hydrophobicities and hydrophobic tag length do not appear to form aggregates in aqueous solution, determined from end-tag W fluorescence spectra showing these W-residues to be in a polar environment (λ_max_ = 358 nm; results not shown).

As demonstrated, W/F-tagging is a facile and flexible approach for reaching increased bactericidal potency for ultra-short AMPs, without the need for post-peptide-synthesis modification, applicable for both Gram-negative and Gram-positive bacteria. Through the composition and/or the length of the hydrophilic peptide and/or the hydrophobic tag, potency and toxicity of the peptide can be tuned, e.g., depending on the relative need of bactericidal potency and limited toxicity. As shown in a previous investigation with longer AMPs, W/F peptide tagging may be applied to a broad range of AMPs, but particularly so to polar and highly charged peptides [28]. This flexibility is attractive from a therapeutic versatility perspective, since it allows one AMP to be further modified to fit the conditions of the indication at hand. Particularly for AMPs not sensitive to infection-related proteolytic degradation, the finding that hydrophobic tagging may be achieved without affecting proteolytic stability also opens up new avenues in applications characterized by high proteolytic activity, such as chronic wounds, eye infections, and cystic fibrosis. The biological relevance of the ultra-short end-tagged peptides was clearly demonstrated here in the skin infection model.

In fact, W/F tagging may possibly be employed to enhance the biological activity of membrane-interacting peptides in a broader perspective. For example, Ember et al. found that hydrophobic tagging increased the biological potency of short C3a-based peptides, and that peptide potency increased with an increasing number of W in the hydrophobic tag [56]. Apart from the effect of the end-tag on membrane interactions, W-tagging was demonstrated to promote specific peptide binding to the C3a receptor. Hence, the binding facility of W-tagged peptides seem to include not only lipid membranes and LPS, as demonstrated in the present investigations, but also more specific targets. Given the potency observed for negatively charged membranes, the end-tagged peptides may potentially also offer opportunities in other contexts, e.g., as homing pro-apoptotic peptides [57].

### Conclusions

End-tagging by W/F stretches strongly enhances the bactericidal potency of ultra-short AMPs, even at physiological ionic strength and in the presence of serum. Hydrophilic peptides as short as 4–7 amino acids long may thus be rendered potent against Gram-negative *E. coli* and Gram-positive *S. aureus*. Although toxicity increases with increasing tag length, compositions could be found at which little or no toxicity is observed, but where the peptides display high bactericidal potency. In contrast to acyl-modified lipopeptides, the present approach facilitates straightforward synthesis of hydrophobically modified AMPs without the need for post-peptide synthesis modifications. The biological relevance of the tagged peptides obtained was demonstrated *ex vivo*. The tagging, which does not detrimentally affect the proteolytic stability of the peptides, promotes peptide binding to bacteria and subsequent wall rupture. Analogously, W-tagging promotes peptide-induced leakage, particularly in anionic, bacteria-mimicing, liposomes, but also LPS binding, both effects probably contributing to the selectivity observed between bacteria and eukaryotic cells.

## Supporting Information

Figure S1 Generalization of the concept of end-tagging by hydrophobic amino acid stretches. Antimicrobial activity as assessed by radial diffusion assay (RDA) against E. coli ATCC 25922 and S. aureus ATCC 29213 of the indicated peptides in absence (open bars) or presence (black bars) of 0.15 M NaCl (mean values are presented, n = 3). “*” denotes no clearence zone detected(A). Effects of peptides on HaCaT cells and erythrocytes in the presence and absence of human serum. The MTT-assay (upper panel) was used to measure viability of HaCaT keratinocytes in the presence of the indicated peptides. In the assay, MTT is modified into a dye, blue formazan, by enzymes associated to metabolic activity. The absorbance of the dye was measured at 550 nm. Cell permeabilizing effects of the indicated peptides (middle panel) were measured by the LDH-based TOX-7 kit. Hemolytic effects (lower panel) of the indicated peptides were also investigated. The cells were incubated with the peptides at 60 mM, while 2% Triton X-100 (Sigma-Aldrich, St. Louis, USA) served as positive control. The absorbance of hemoglobin release was measured at 540 nm and is expressed as % of Triton X-100 induced hemolysis (mean values are presented, n = 3) (B). (For MTT and LDH, the difference between tagged and non-tagged peptides is statistically significant in the absence of serum (P<0.001, one way ANOVA), whereas the difference in the presence of serum is not statistically significant. The difference between tagged and non-tagged peptides is not statistically significant regarding hemolysis.)
(2.88 MB TIF)
