# Supplementary figures and images for: End-Tagging of Ultra-Short Antimicrobial Peptides by W/F Stretches to Facilitate Bacterial Killing
Source: PLoS One. 2009 Apr 17;4(4):e5285. doi: 10.1371/journal.pone.0005285 (PMC2667214; doi:10.1371/journal.pone.0005285)

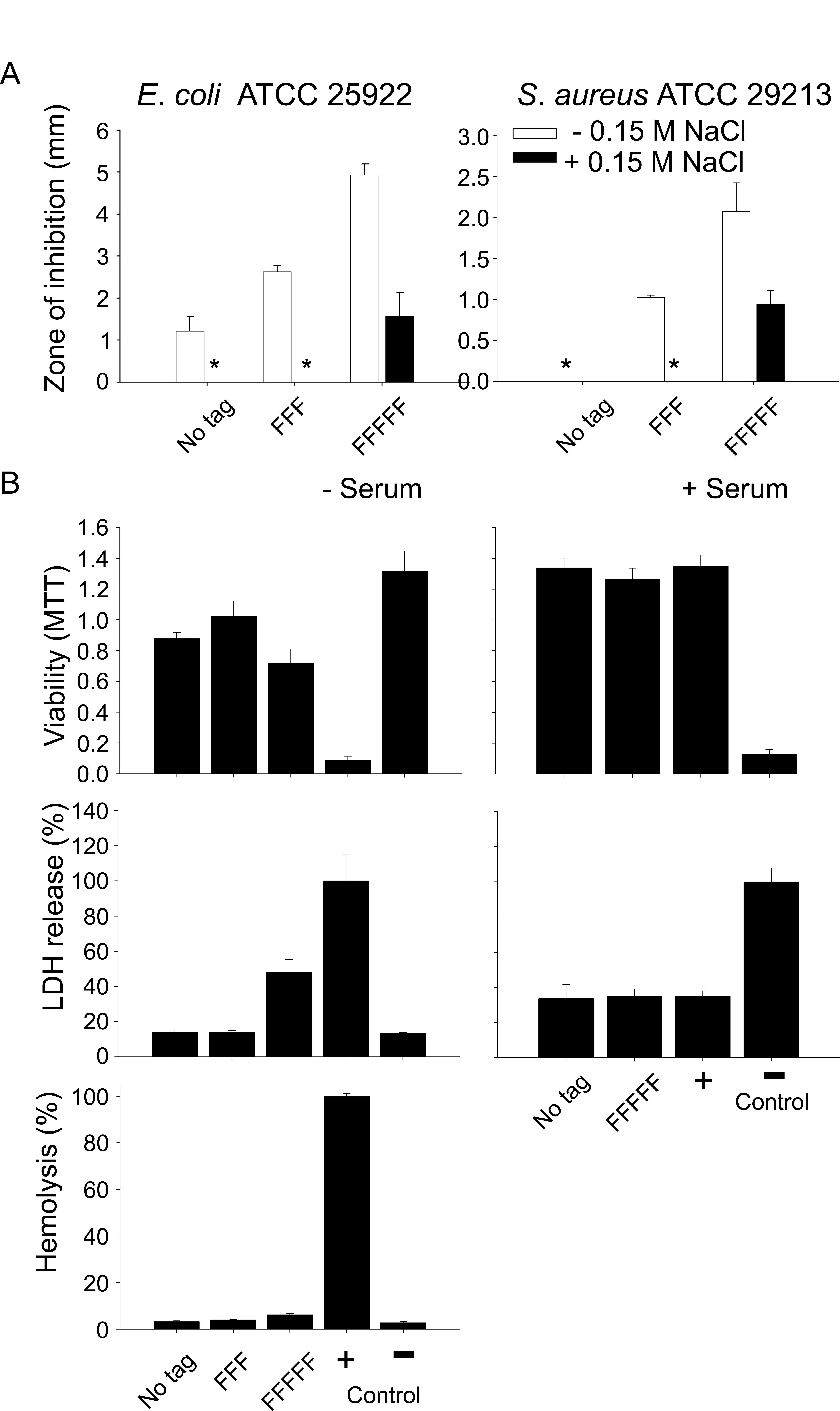

Supplement: Figure S1 — Generalization of the concept of end-tagging by hydrophobic amino acid stretches. Antimicrobial activity as assessed by radial diffusion assay (RDA) against E. coli ATCC 25922 and S. aureus ATCC 29213 of the indicated peptides in absence (open bars) or presence (black bars) of 0.15 M NaCl (mean values are presented, n = 3). “*” denotes no clearence zone detected(A). Effects of peptides on HaCaT cells and erythrocytes in the presence and absence of human serum. The MTT-assay (upper panel) was used to measure viability of HaCaT keratinocytes in the presence of the indicated peptides. In the assay, MTT is modified into a dye, blue formazan, by enzymes associated to metabolic activity. The absorbance of the dye was measured at 550 nm. Cell permeabilizing effects of the indicated peptides (middle panel) were measured by the LDH-based TOX-7 kit. Hemolytic effects (lower panel) of the indicated peptides were also investigated. The cells were incubated with the peptides at 60 mM, while 2% Triton X-100 (Sigma-Aldrich, St. Louis, USA) served as positive control. The absorbance of hemoglobin release was measured at 540 nm and is expressed as % of Triton X-100 induced hemolysis (mean values are presented, n = 3) (B). (For MTT and LDH, the difference between tagged and non-tagged peptides is statistically significant in the absence of serum (P<0.001, one way ANOVA), whereas the difference in the presence of serum is not statistically significant. The difference between tagged and non-tagged peptides is not statistically significant regarding hemolysis.) (2.88 MB TIF) [file pone.0005285.s001.tif]
